# Counselling services embedded within rheumatology clinics could help bridge the gap in mental health care provision for adults with rheumatic diseases

**DOI:** 10.1093/rap/rkac080

**Published:** 2022-10-10

**Authors:** Georgia Hayward, Rachel Mandela, Andrew Barr, Jane Freeston, Claire Vandevelde, Helena Marzo-Ortega

**Affiliations:** Rheumatology Department, Leeds Teaching Hospitals Trust, Leeds, UK; Clinical Psychology Department, Leeds Teaching Hospitals Trust, Leeds, UK; Rheumatology Department, Leeds Teaching Hospitals Trust, Leeds, UK; NIHR Leeds Biomedical Research Centre, Leeds Teaching Hospitals NHS Trust, Leeds, UK; Leeds Institute of Rheumatic and Musculoskeletal Medicine, University of Leeds, Leeds, UK; Rheumatology Department, Leeds Teaching Hospitals Trust, Leeds, UK; NIHR Leeds Biomedical Research Centre, Leeds Teaching Hospitals NHS Trust, Leeds, UK; Leeds Institute of Rheumatic and Musculoskeletal Medicine, University of Leeds, Leeds, UK; Rheumatology Department, Leeds Teaching Hospitals Trust, Leeds, UK; NIHR Leeds Biomedical Research Centre, Leeds Teaching Hospitals NHS Trust, Leeds, UK; Leeds Institute of Rheumatic and Musculoskeletal Medicine, University of Leeds, Leeds, UK; Rheumatology Department, Leeds Teaching Hospitals Trust, Leeds, UK; NIHR Leeds Biomedical Research Centre, Leeds Teaching Hospitals NHS Trust, Leeds, UK; Leeds Institute of Rheumatic and Musculoskeletal Medicine, University of Leeds, Leeds, UK

Key message

- Counselling services in rheumatology clinics could help provide mental health support for people with RMDs.

Dear Editor, People with rheumatic musculoskeletal diseases (RMDs), including SpA, have a high prevalence of anxiety and depression [1, 2]. Indeed, there exists a complex relationship between chronic disease and mental disorders whereby both conditions can mutually influence each other, eventually leading to the aggravation of both. Poor adjustment to illness, low self-esteem and independence, depression and anxiety can translate into poor treatment adherence and repeated consultations with health-care professionals [3, 4]. Recognizing this need, the National Health Service (NHS) long-term plan outlined mental health as a priority [5], and the National Institute for Health and Care Excellence (NICE) recommended that psychological interventions be offered to patients with RMDs [6, 7]. Although it might be widely accepted that psychological input should be part of their regular care, the challenge remains to engage managers and commissioners in order to ensure timely access to specialized mental health support for people affected with spondyloarthritis (SpA) and other inflammatory disorders.

We have previously reported our experience of significant psychosocial morbidity associated with RMDs in a tertiary rheumatology centre and that this can be addressed successfully with a short intervention with a specialized clinical psychology team [8]. In our cohort, we observed a decreased demand for rheumatology clinic appointments, which suggests further additional benefits of a clinical psychologist working within the rheumatology setting [8]. However, these interventions often come late, once mental illness has developed, requiring costly long-term management. Access to support, such as counselling and self-management strategies, has the potential to reduce the impact of long-term conditions not only on the patient but on health-care systems and wider society.

Prompted by previous patient feedback on the need to improve our holistic care provision, we sought to explore their opinion on how our service currently supports their mental well-being and whether a counselling service would be a beneficial addition to their care. To this end, consecutive patients attending the adult rheumatology clinics in our Trust were invited to complete a short paper survey comprising five closed and two open-ended questions (Supplementary Table S1, available at *Rheumatology Advances in Practice* online) over 10 days in May 2021. This survey was performed as part of an NHS service improvement project based on patient input. No ethical approval was required, and patients gave their implicit consent by taking part in the survey.

Seventy-three (50% SpA) participants responded to at least one question. On a Likert scale, ranging from well supported (score 10) to no support (score 0), 48% (of 62 respondents) felt that their mental health was not adequately supported (scoring ≤5). Most participants (66%) indicated that they would find it useful to speak to someone about the impact of their condition, with 78% acknowledging that there were times in the past when this would have been useful.

Responses to the two open-ended questions were clustered into six themes: accessibility (e.g. counselling availability in clinic); information (e.g. about counselling services); empathic listening and staff awareness; support for pain; routine screening/checks; and improved access to medical/nursing staff for condition management. Overall, 67% of participants agreed that meeting the counsellor when attending their rheumatology clinic appointment would make them more likely to use a counselling service. The majority (75%, *n* = 73 respondents) reported that they would feel confident to disclose a struggle in their mental well-being with the rheumatology team.

These results suggest that, despite improved access to a dedicated clinical psychology service, we are not adequately supporting the psychological needs of patients, with more than half of those surveyed stating that they would find it beneficial to talk with someone about the impact of their RMD. It was evident from the answers collected that patients would find it valuable to have a regular check or for their mental health to be screened routinely. Importantly, our patients would like their clinical team to have an awareness of how their medical condition can affect their mental health and vice versa, including pain. In particular, patients appreciate empathy and want this to be conveyed by professionals in their consultations. The majority of participants would have valued a counselling service being available to them, with more than half stating that meeting the counsellor in clinic would positively influence them in using a counselling service.

These observations open up a new way to address RMDs with a more holistic approach, in which mental health needs are considered alongside physical manifestations of organic disease. Having easy access to counselling services, particularly at the time of diagnosis and/or the early disease stage, is likely to help individuals achieve more control over their own health by increasing their understanding and acceptance of their chronic condition and the care they receive.

In conclusion, mental health support remains an unmet need in RMDs. People living with these conditions want this to be addressed and would value opportunities to access counselling services while attending routine rheumatology appointments. Further research is needed to show the potential impact of this type of intervention, particularly at the time of diagnosis and in early disease, when a short-lived intervention with a specialist counsellor might negate the need for formal psychological or even psychiatric support at a later stage.

## Supplementary data

Supplementary data are available at *Rheumatology Advances in Practice* online.

## Supplementary Material

rkac080_Supplementary_Data

## Data Availability

Data are available upon reasonable request. All data relevant to the study are included in the article.
