# Supplementary material for: Counselling services embedded within rheumatology clinics could help bridge the gap in mental health care provision for adults with rheumatic diseases
Source: Rheumatol Adv Pract. 2022 Oct 10;6(3):rkac080. doi: 10.1093/rap/rkac080 (PMC9580513; doi:10.1093/rap/rkac080)
Supplement: rkac080_Supplementary_Data [file rkac080_supplementary_data.docx]

| **Supplementary Table S1. Survey Questions** | |
| --- | --- |
| **Question Number** | **Question** |
| 1 | How well does the rheumatology service support your mental health? 10 point likert scale, 0 (not supported) to 10 very supported |
| 2 | Would you find it useful to speak to someone about the impact of your rheumatological condition on your mental health? Yes or No |
| 3 | Have there been times in the past when you think it might have been helpful to speak to someone about the impact of your rheumatological condition? Yes or No |
| 4 | If you felt you were struggling with your mental well-being, would you feel confident to share this with the team in your appointment? Yes or No |
| 5 | What would make you more likely to access a counselling service? open ended |
| 6 | What do you think the service could do to better support your mental health? open ended |
| 7 | Would meeting the counsellor in clinic for an informal chat make you more likely to use this service? Yes or No |
